# A simple joint control pattern dominates performance of unconstrained arm movements of daily living tasks

**DOI:** 10.1371/journal.pone.0235813

**Published:** 2020-07-13

**Authors:** Natalia Dounskaia, Yury Shimansky, Bryan K. Ganter, Meghan E. Vidt

**Affiliations:** 1 Arizona State University, Kinesiology Program, Phoenix, AZ, United States of America; 2 Mayo Clinic, Scottsdale, AZ, United States of America; 3 Biomedical Engineering, Pennsylvania State University, University Park, PA, United States of America; 4 Penn State College of Medicine, Physical Medicine and Rehabilitation, Hershey, PA, United States of America; Georgia State University, UNITED STATES

## Abstract

A trailing joint control pattern, during which a single joint is rotated actively and the mechanical effect of this motion is used to move the other joints, was previously observed during simplified, laboratory-based tasks. We examined whether this simple pattern also underlies control of complex, unconstrained arm movements of daily activities. Six tasks were analyzed. Using kinematic data, we estimated motion of 7 degrees of freedom (DOF) of the shoulder, elbow, and wrist, and the contribution of muscle and passive interaction and gravitational torques to net torque at each joint. Despite task variety, the hand was transported predominantly by shoulder and elbow flexion/extension, although shoulder external/internal rotation also contributed in some tasks. The other DOF were used to orient the hand in space. The trailing pattern represented by production of net torque by passive torques at the shoulder or elbow or both was observed during the biggest portion of each movement. Net torque generation by muscle torque at both joints simultaneously was mainly limited to movement initiation toward the targets and movement termination when returning to the initial position, and associated with needing to overcome gravity. The results support the interpretation of previous studies that prevalence of the trailing pattern is a feature of skillful, coordinated movements. The simplicity of the trailing pattern is promising for quantification of dyscoordination caused by motor disorders and formulation of straightforward instructions to facilitate rehabilitation and motor learning.

## Introduction

Motor disorders often cause dyscoordination of arm movements [1–5]. To understand the effect of each disorder on movement performance, normal coordination of joint motions during multi-joint movements needs to be understood. Although motor tasks are usually formulated in terms of hand motion, they are performed through proper joint coordination. For example, reaching straight to a target and drawing a circle are achieved through certain coordinated motion of the arm’s joints. However, the role of joint coordination is not limited to performance of tasks. This is suggested by findings that during development of a new motor skill, a hand movement that achieves the task goal is acquired early in practice, while joint coordination continues to change [6, 7]. Thus, joint coordination is an implicit part of the movement control goal. Knowing how the limb joints are coordinated during skillful movements would enable explicit training of joint coordination, which may enhance effectiveness of rehabilitation and motor learning procedures.

The importance of studying joint coordination becomes more evident when we take into account complex dynamics influencing joint motions. Net torque (NT) that causes acceleration/deceleration of a joint is a sum of several torques. In addition to active control represented by muscle torque (MT), at least two passive torques also influence motion of each joint [8]. These are gravitational torque (GT) caused by gravity and interaction torque (IT) caused by mechanical interactions of the inter-connected limb segments. Considering total passive torque (PT = GT + IT) results in the relation NT = MT + PT. This equation shows that MT needs to be adjusted to PT to result in required NT. Furthermore, PT at each joint depends on current angular positions, velocities, and accelerations at the other joints of the limb. Joint coordination can therefore be viewed as the adjustment of MT to PT across all joints of the limb. Thus, to understand control of coordinated multi-joint movements, we need to understand how the central nervous system (CNS) determines MT for all joints of the limb in the presence of complex and variable PT.

A possible interpretation is offered by experimental observations of a “trailing” joint control pattern that was revealed during various arm movements [9–30]. The trailing pattern usually consists in the use of a single (leading) joint to generate the mechanical foundation for motion of the other (trailing) joints of the limb, analogous to motion of a whip handle which brings into motion the entire cord. In contrast to the passive cord of a whip, musculature at the trailing joints can interfere and modify their passive motion. Sometimes, when gravity can be used to produce energy for limb motion, the leading joint is not needed, and all joints are driven predominantly passively [31].

The trailing pattern was usually revealed through torque analysis, although the difference in control between the leading and trailing joints has been verified with electromyography [12, 13, 29, 30]. Usually, the relation NT = MT + PT is used to estimate the relative contribution of MT and PT to NT at each limb’s joint. Despite the large variety of the studied tasks, there was typically a single joint that was rotated predominantly actively, i.e., at this joint, NT was mainly caused by MT while PT was either small or opposite in sign (resistive) to NT. In contrast, control of the remaining joints was most often organized in one of two ways. The first way was predominantly passive rotation of the joint, i.e., NT was produced mainly by PT with MT being small or resistive to NT. The second way was near fixation of the joint, i.e., PT and MT largely cancelled each other, resulting in low NT. One study of shoulder and elbow movements also reported the generation of NT predominantly by GT at both joints when the arm moved downward [31].

The consistent observation of this organization of joint control prompted the leading joint hypothesis according to which the brain uses a strategy for control of multi-joint movements that exploits mechanical factors acting at the joints of the multi-segmental limbs [32, 33]. Since GT and IT depend on limb motion, this strategy consists of moving the leading joint in a way that causes passive motion at the remaining joints (through motion-dependent IT and GT) which, with modest adjustments of it by MT, results in the motion of the entire limb that accomplishes a given motor task. The advantage of this control strategy is that it simplifies joint coordination. Indeed, the trailing joints are coordinated with the leading joint predominantly passively, by PT. If a trailing joint is fixed, the maintenance of the constant angle between the proximal and distal limb segments does not require coordination with motion of the other joints. A formal justification of simplification of joint coordination through the trailing pattern is presented elsewhere [34].

The nearly passive motion of the trailing joint is not necessarily observed during all multi-joint movements. For example, during horizontal arm movements (GT = 0) that require the shoulder and elbow to simultaneously extend, NT at both joints is generated mainly by MT because IT resists rotation of both joints [12, 20]. Rather, the trailing pattern is the preferred joint control pattern that is used when the task and conditions allow it [19, 35, 36]. The preference to use this pattern suggests that it represents optimal joint coordination. Indeed, movements of experts are characterized by a more pronounced trailing pattern (i.e., by a higher contribution to NT of MT at the leading joint and of PT at the trailing joints) compared with novices [16, 37]. Also, joint dyscoordination during movements of the nondominant arm and in patients with neurological disorders, including Parkinson’s disease, stroke, deafferentation, and developmental coordination disorder, is associated with a reduced ability to exploit PT for movement production represented by deteriorations in the trailing pattern [3, 5, 38–42].

While the use of the trailing joint control pattern has been supported by multiple studies, they were predominantly limited to simplified, laboratory-based movements that accomplished artificial motor tasks and tasks that were restricted to a plane either for motion of the entire arm [e.g., 12, 15–17, 20, 25, 37, 43, 44] or the hand [31, 35, 45]. It is important to examine whether the trailing pattern is also used during natural, unconstrained, arm movements of daily activities. There are factors that may cause differences in joint control between simplified laboratory movements and unconstrained movements of daily activities. The latter are usually not fast, which increases the influence of position-dependent GT compared with velocity- and acceleration-dependent IT. This may change the way PT is incorporated in joint control. Also, activities of daily living involve three-dimensional (3D) arm movements with minimal restrictions on hand trajectories, and therefore, they are characterized by high redundancy of kinematic degrees of freedom (DOF), which allows performance of each task through many different joint coordination patterns. The DOF redundancy increases the opportunity for using the preferred trailing pattern. We therefore hypothesized that the trailing pattern dominates control of daily arm movements.

To test this hypothesis, we used tasks that replicated arm movements performed during representative activities of daily living (ADL). Revealing the trailing pattern would directly inform rehabilitative approaches on how these ADL movements can be evaluated and trained in special populations. Specifically, we used 6 ADL tasks: placing a cup on a shelf of 2 different heights, hair combing, bringing a cup to the mouth for drinking, turning a book page, and placing the hand on the chest like in a pledge, which is often needed during dressing. We selected these tasks because they represent frequently performed daily activities and provide a variety of joint control patterns by requiring movements in different planes and directions, different involvements of the arm’s DOF, and different initial and final arm postures. For example, the hand moved away from the body during the reaching tasks, to the middle of the body at different heights during drinking and hair combing, and across the body at different heights during pledge and page turning. Due to demands of the torque analysis methods used here, we selected tasks during performance of which the environment did not apply any substantial forces to the arm besides gravitation. Most of the selected tasks required lifting the hand up. To consider examples of downward movements, we included in the analysis the returning movements to the initial position.

## Methods

### Subjects

Fourteen right-handed young adults (7 males and 7 females, 21.7 ± 2.2 years of age) were recruited from the Arizona State University community to participate in this study. Handedness was verified through a questionnaire adapted from the Edinburgh Handedness Inventory [46]. All participants had no known neurological or musculoskeletal impairments. All participants provided written informed consent prior to participating in the study. The Institutional Review Board at Arizona State University approved the experimental protocol.

### Procedure and design

**Fig 1:**
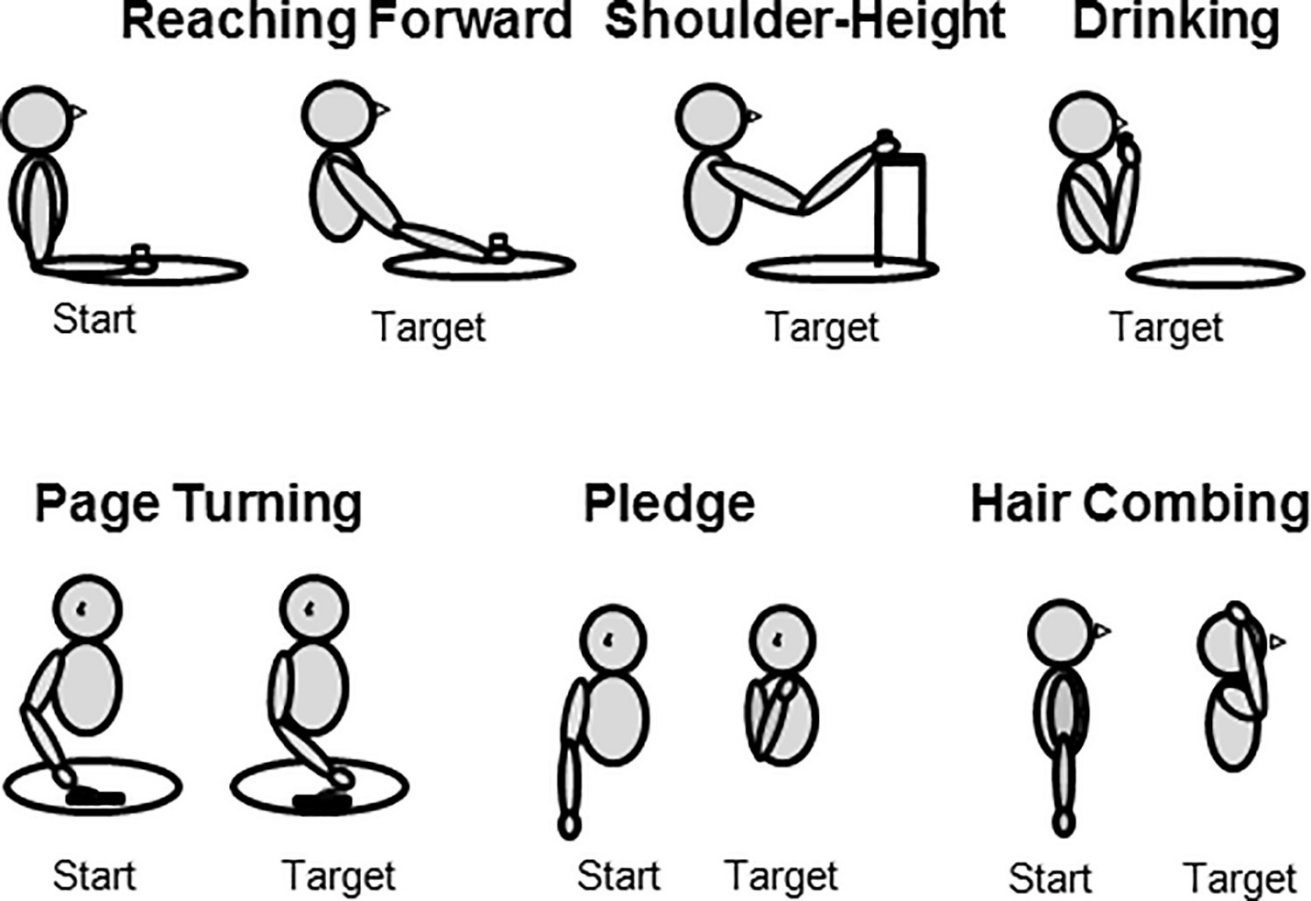
A schematic representation of the initial and final position of the arm during the 6 tasks. The top panels show the starting arm posture and target postures for the forward reach, shoulder-level reach, and drinking. The bottom panels show the starting and target arm postures for hair combing, pledge, and page turning.

Participants performed 6 functional movement tasks that represented typical ADL (Fig 1). Four of these tasks were performed while seated on a chair and 2 tasks were performed while seated on a stool without a back. The tasks included in the first set were:

- page turning,
- drinking (bringing an empty soda can to the mouth for drinking),
- 2 reaching tasks during which an empty soda can was moved forward and to shoulder height.

The second set included:

- hair combing,
- pledge (bringing the hand to the left side of the chest often performed during self-care).

During the 4 tasks of the first set, participants were seated on a chair in front of a height-adjustable table. Participants were strapped to the chair back to minimize trunk movement and limit motion to the arm only. In the initial position, the hand was placed on the table so that the wrist was at the table’s edge, the upper arm was hanging down so that its projection on the sagittal plane was vertical, the forearm was horizontal, and the hand was aligned with the forearm (see the Start posture in the top-left corner of Fig 1). During the reaching and drinking tasks, participants held an empty soda can that was resting on the table. The initial position of the can was marked on the table with a piece of tape to indicate where the can needed to be returned after the task was completed.

During the 2 reaching tasks, the reach distances were individualized for each participant, using 80% of the length of the participant’s forearm at the target position [47]. The selected target distance did not require full extension of the elbow in both reaching tasks, thus minimizing passive elastic torque at the elbow that becomes substantial near anatomical limits of the joints. The individualized approach was necessary to minimize the influence of differences in anthropometric characteristics of subjects on joint motions and torques exerted at the joints. During reaching forward, the target position was on the table, straight away from the initial position of the can in the anterior direction, and it was marked with a 1 cm x 1 cm piece of tape. Participants lifted the can from the table, moved it to the target position, placed it on the table with the middle of the can on the tape mark, and then returned the can to the initial position. During shoulder-height reaching, a shelf of the corresponding, individually adjusted height was positioned at the target distance, and the participant placed the can on the shelf in front of the right shoulder. Accuracy at the target and movement start was de-emphasized. During the drinking task, participants brought the can to their mouth to simulate drinking. During the page turning task, a large opened textbook was positioned on the table. The right bottom corner of the book was in front of the right shoulder at the same starting location as that used in the other tasks. Participants held a few pages at the right bottom corner of the book, turned these pages to the left, and then turned the pages back to the initial position.

The 2 tasks of the second set, hair combing and pledge, were performed while participants were seated on a stool without a back with no table in front of them. This provided a condition in which the table and chair back did not restrict arm motion during the tasks. Participants were instructed to keep the trunk motionless and limit motion to the arm only. The arm initially was hanging down freely by the side of the trunk. The pledge task consisted of bringing the right hand across the body and placing it upon the left side of the chest. During the hair combing task, participants held a hair comb in the right hand. Participants lifted the hair comb to the front of their head, made one front-back hair combing motion, and returned the arm to the initial position. Participants were instructed to perform the entire movement smoothly, without stopping.

In all tasks, each movement was initiated by a verbal “go” command. Participants were instructed to pause for 2–3 seconds at the target position to assure that the hand completely stopped and then to return the arm to the initial position. An exception was the hair combing task, during which subjects did not stop the hand at the back of their head before returning to the initial position. Participants were able to self-select their arm movement trajectories between the instructed start and end positions during the performance of each task. All tasks were performed at comfortable, self-selected speed. For each task, participants performed 5 trials, the first 2 of which were practice trials, while the last 3 trials were recorded. To prevent fatigue, a rest period of at least 30 seconds was provided between trials and tasks. Although 42 trials of each task were recorded from the 14 subjects, only 36 trials of reaching forward were available for analysis due to an error in motion capture in all 6 trials recorded from 2 subjects. Also, 41 trials were successfully captured for the drinking task. All 42 trials were used for the other 4 tasks.

### Data collection and determination of the arm’s degrees of freedom

**Fig 2:**
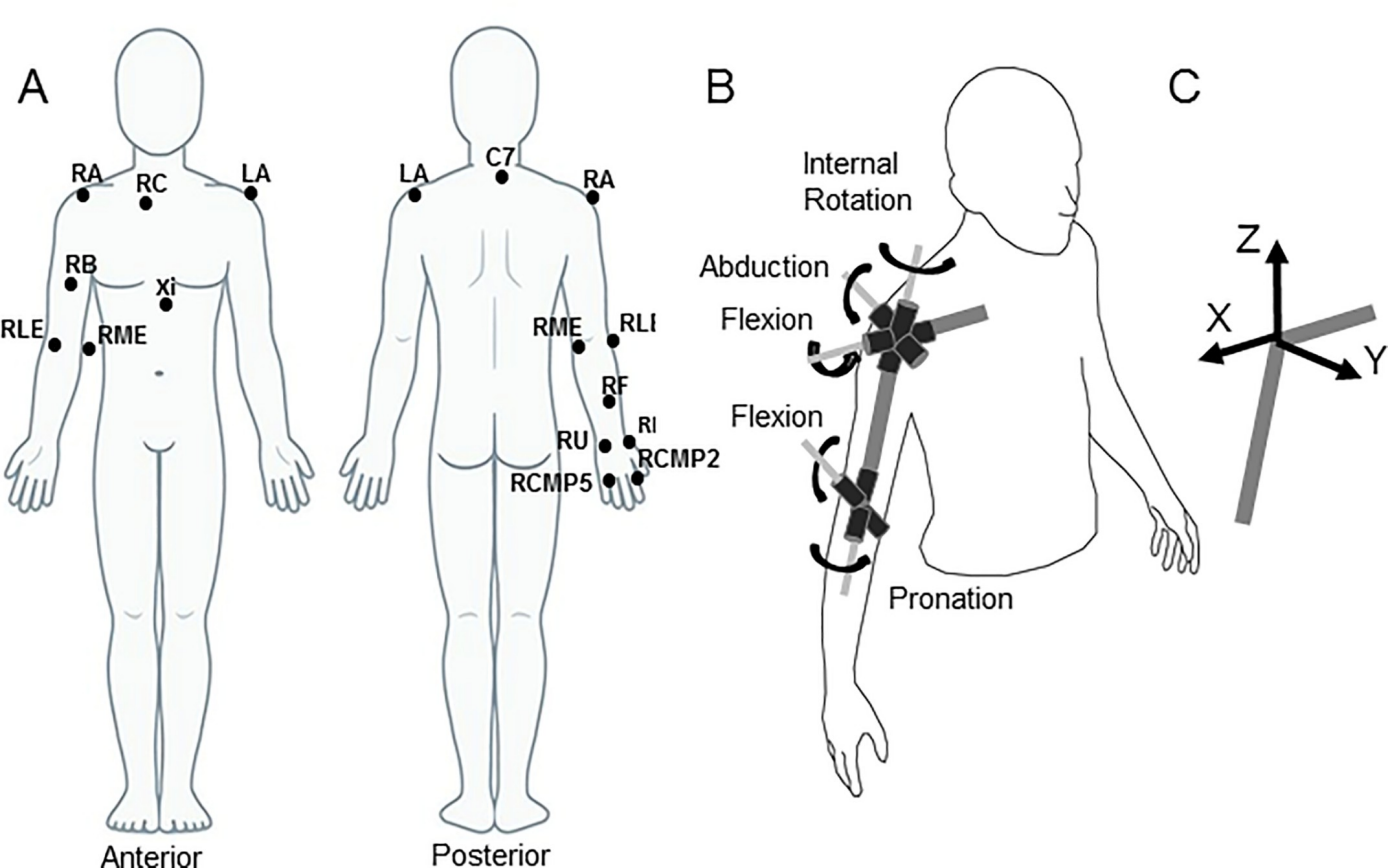
Experimental procedures. **A**: Locations of the reflective markers. C7: 7^th^ cervical vertebra; RA: Right acromion; LA: Left acromion; RC: Right clavicle; Xi: Xiphoid process; RB: Right biceps; RLE: Right lateral epicondyle; RME: Right medial epicondyle; RF: Right forearm; RR: Right radial styloid; RU: Right ulnar styloid; RCMP2: Right 2^nd^ metacarpophalangeal joint; RCMP5: Right 5^th^ metacarpophalangeal joint. **B**: Definition of the 3 shoulder DOF (internal/external rotation, abduction/adduction, and flexion/extension) and 2 elbow DOF (elbow flexion/extension and forearm pronation/supination). **C**: The global coordinate system was centered in the shoulder joint. The X-axis was directed laterally to the right, the Y-axis was directed anteriorly, and the Z-axis was directed vertically upward.

Arm motion was recorded using Cortex Version 6.0 software (Motion Analysis Corporation, Santa Rosa, CA). Eight Kestrel motion capture cameras (Motion Analysis Corp., Santa Rosa, CA), with accuracy to 0.8mm, were placed surrounding the workspace. Prior to data collection, the system was calibrated as per manufacturer’s instructions. The motion of thirteen 1 cm-diameter retro-reflective markers was tracked at 200Hz using Cortex software (v.6.0, Motion Analysis Corp., Santa Rosa, CA). Markers were placed on anatomical locations on the trunk and right arm: the second metacarpophalangeal joint (RCMP2), fifth metacarpophalangeal joint (RCMP5), radial styloid (RR), ulnar styloid (RU), forearm (RF), medial epicondyle (RME), lateral epicondyle (RLE), head of the biceps brachii (RB), head of right clavicle (RC), right acromion (RA), left acromion (LA), seventh cervical vertebra (C7), and xiphoid process (Xi) (Fig 2A). Standard post-processing of marker data was performed with Cortex software that determined a global 3D coordinate system, identified each marker through each trial, and virtually recreated marker frames with missing points. Following previous studies of arm movements [e.g., 36, 48] marker data were filtered using a 7 Hz low-pass dual-pass 4^th^-order Butterworth digital filter. The centers of the elbow and wrist joints and the hand were computed as a midpoint of the markers RLE and RME for the elbow, RR and RU for the wrist, and RMCP2 and RMCP5 for the hand.

The joint position data were used to assess motion at the 7 DOF of the arm, using an original methodology [49]. The 7 DOF and their positive and negative directions were: shoulder internal (+) and external (-) rotation (rotation about the longitudinal axis of the humerus), shoulder abduction (+) and adduction (-), shoulder flexion (+) and extension (-), elbow flexion (+) and extension (-), forearm pronation (+) and supination (-), wrist flexion (+) and extension (-), wrist radial (+) and ulnar (-) deviation. The shoulder and elbow DOF are shown in Fig 2B.

The global coordinate system in which data coordinates were computed had the origin at the shoulder joint and its axes were directed in the lateral, anterior, and vertical upward directions as shown in Fig 2C. According to the used method [49], the 3D coordinates of the joint centers and markers RA and LA were used to build relative joint coordinate systems at the shoulder, elbow, and wrist. The 3 axes of each relative coordinate system represented axes of rotation at the anatomical DOF of the corresponding joint. The relative coordinate systems were used to compute a 3D vector of angular velocity at each joint. The angular velocity vectors were used to conduct kinematic and kinetic analyses. Both types of analyses were similar to those we conducted previously [31, 36, 45], and therefore, they are presented next only briefly.

### Kinematic analysis

Translational hand velocity was calculated for each trial as a derivative of the time series of the distance covered by the hand between each pair of data points. Each trial included movement from the initial position to the target and movement from the target back to the initial position. The hand velocity profile therefore had 2 pronounced peaks separated by a short period of zero values when the hand dwelled in the target position. The beginning of the movement to the target was determined as the time moment when the hand velocity exceeded 5% of the value of the first peak [20]. Accordingly, the movement end was determined as the time moment when the hand velocity decreased below 5% of the peak value. The beginning and end of the returning movement were determined in the same way using the second velocity peak. In some trials, especially during hair combing, hand velocity decreased between the 2 peaks but did not reach zero. Then the time moment of minimal velocity between the 2 peaks was used as the end of the movement to the target and beginning of the returning movement.

To assess the amount of motion at each DOF, DOF angular excursion was computed at each time step as the integral of the angular velocity at this DOF between the movement beginning and the current time step. DOF excursion amplitudes were computed as the difference between the final and initial DOF angles. In addition, the contribution of each DOF to hand velocity was computed with the use of the following expression for hand velocity magnitude [36, 49, 50]:

$$\left|v\right|=\sum_{i=1}^{7}\left(v_{i}\cdot v_{u}\right),$$ (1)

where **v** is the vector of hand velocity, **v**_*u*_ is its unit vector, and **v**_*i*_ is the vector of hand velocity produced by rotation at the *i*^th^ DOF. The latter is computed as **v**_*i*_ = **w**_*i*_ x **p**_*i*_, where **w**_*i*_ is the angular velocity vector at the DOF and **p**_*i*_ is the vector from the joint center to the hand center. We used the procedures presented in [49] to compute **w**_*i*_ and **p**_*i*_. According to *Eq* 1, the contribution of DOF *i* to the production of the hand velocity is represented by the projection of **v**_*i*_ on the unit vector **v**_*u*_.

### Kinetic analysis

Torques at the shoulder, elbow, and wrist were computed at each time step as 3D vectors, using published equations [49]. The equations represent an inverse dynamics model that includes the trunk, upper arm, forearm, and hand connected to each other through the shoulder, elbow, and wrist joints. The trunk is assumed motionless. The equations use as an input the angular velocities at the 7 DOF of the 3 joints and accelerations that were computed as derivatives of velocities. They also use mass and length of each arm segment (upper arm, forearm, and hand), and distance from the segment’s center of mass to the segment’s proximal joint. These anthropometric characteristics were estimated from the weight and height of each participant, using regression equations [51]. The output of the equations included 3D vectors of net torque (NT), interaction torque (IT), and gravitational torque (GT) at each joint. Muscle torque (MT) was computed as a vector difference **MT** = **NT**–**IT**–**GT**. The bold font signifies vector quantities. **NT** causes rotation at the joint. **IT** is passive torque that emerges due to mechanical interactions among the limb’s segments. **GT** is passive torque caused by gravitation. **MT** largely represents active control produced through muscle activation, although it also includes passive torques due to elasticity of tissues surrounding the joint. However, these passive elastic torques are low if joint motion does not approach its anatomical limits [8, 52]. The passive component of **MT** was minimal in our experiment because the initial arm position and movement target were selected to assure that the arm’s joints would not approach their anatomical limits during any of the studied movements.

The purpose of the kinetic analysis was to assess the role of **MT**, **IT**, and **GT** in generation of **NT** at each joint. This was achieved by considering projections **MT**_**NT**_, **IT**_**NT**_, and **GT**_**NT**_ of the 3 torque vectors on the vector **NT**. The projection magnitude (MT_NT_, IT_NT_, GT_NT_) was considered positive if the projection had the same direction as **NT** and negative if the direction of the projection was opposite to that of **NT**. Thus, the sign of each projection magnitude indicated whether the corresponding torque contributed to or resisted generation of **NT**. The projections were used to quantify relative contribution of **MT** and total passive torque (**PT** = **IT** + **GT**) to **NT**. Namely, a muscle torque contribution (MTC) to **NT** was calculated at each moment of time as [36]:

$$MTC=\left\{ \begin{array}{c} {MT}_{NT}/NT\;if\;0<{MT}_{NT}<NT,\\ 1\;if\;{MT}_{NT}\geq NT,\\ 0\;if\;{MT}_{NT}<0. \end{array}\right.$$ (2)

MTC = 1 when **MT**_**NT**_ and **NT** have the same direction and magnitude MT_NT_ exceeds magnitude NT. MTC = 1 signifies the control pattern during which **MT** generated entire **NT** and also suppressed **PT** which resisted joint motion. We use wording “**NT** was actively generated” in this case. MTC = 0 when **MT**_**NT**_ is opposite in direction to **NT**. MTC = 0 signifies that **NT** was generated by **PT** and **MT** resisted joint motion. The wording we use in this case is: “**NT** was passively generated”, even though MT could be substantial. Values of MTC between 0 and 1 indicate that both **MT** and **PT** contributed to generation of **NT,** with higher values signifying a greater contribution of **MT** compared with **PT**. MTC was used to separate periods of joint motion during which the joint was rotated predominantly actively (MTC ≥ 0.5) and predominantly passively (MTC < 0.5).

## Results

Next, results for movements to the targets are presented. Characteristics of the returning movements are presented at the end of the Results section.

### Hand motion

Movement time and trajectory length differed across the 6 tasks (the top of Table 1). Both characteristics were the shortest during reaching forward. Page turning took the longest time to perform and hair combing had the longest hand trajectory.

**Table 1:** Mean and SD of movement characteristics of the hand and 7 DOF for each task*.

	Forward Reach	Shoulder-Level Reach	Hair Combing	Drinking	Pledge	Page Turning
**Hand Movement Characteristics**						
Mov. Time (sec)	0.88 (0.16)	0.93 (0.18)	1.18 (0.23)	1.00 (0.27)	1.05 (0.19)	1.23 (0.25)
Traj. Length (cm)	24 (4)	42 (4)	116 (8)	42 (7)	88 (11)	53 (11)
**DOF Amplitudes (°)**						
Shoulder Flex/Ext	32 (6)	51 (7)	79 (11)	42 (15)	24 (13)	26 (10)
Shoulder Abd/Add	9 (8)	6 (7)	33 (9)	5 (5)	5 (12)	-8 (5)
Shoulder Int/Ext Rot.	10 (10)	-2 (8)	-12 (22)	10 (10)	60 (23)	56 (15)
Elbow Flex/Ext	-37 (20)	-36 (10)	87 (11)	28 (9)	90 (28)	-14 (11)
Forearm Pron/Supin	-10 (12)	-9 (7)	-67 (23)	7 (8)	-48 (20)	25 (12)
Wrist Flex/Ext	2 (7)	-8 (4)	-4 (9)	1 (5)	4 (9)	-3 (6)
Wrist Rad/Uln Dev.	-7 (9)	0 (7)	3 (9)	-1 (6)	13 (10)	11 (12)
**Ratio of Inter- and Intra-Subject Variability**						
Min across 7 DOF	1.7	1.8	1.4	2.8	2.9	2.1
Max across 7 DOF	4.3	6.0	2.5	8.2	4.9	5.1
**DOF Contributions to Hand Velocity (%)**						
Shoulder Flex/Ext	37 (15)	42 (11)	37 (10)	43 (18)	8 (12)	21(7)
Shoulder Abd/Add	8 (5)	7 (6)	3 (6)	-0 (2)	1 (8)	5 (4)
Shoulder Int/Ext Rot.	7 (11)	5 (5)	13 (7)	7 (5)	30 (11)	40 (8)
Elbow Flex/Ext	43 (13)	38 (14)	40 (13)	41 (20)	56 (15)	26 (8)
Forearm Pron/Supin	-1 (1)	-1 (2)	0 (1)	0 (1)	1 (2)	-1 (2)
Wrist Flex/Ext	-2 (2)	1 (2)	1 (2)	0 (2)	1 (1)	-0 (2)
Wrist Rad/Uln Rot.	2 (2)	0 (1)	1 (2)	-1(1)	1 (1)	0 (1)
**Percentage of Hand Velocity Accounted by DOF Contributions (%)**						
	93 (9)	91 (7)	94 (4)	90 (6)	97 (3)	90 (7)

* The means and SD values were computed across all movements of each type performed by all subjects.

**Fig 3:**
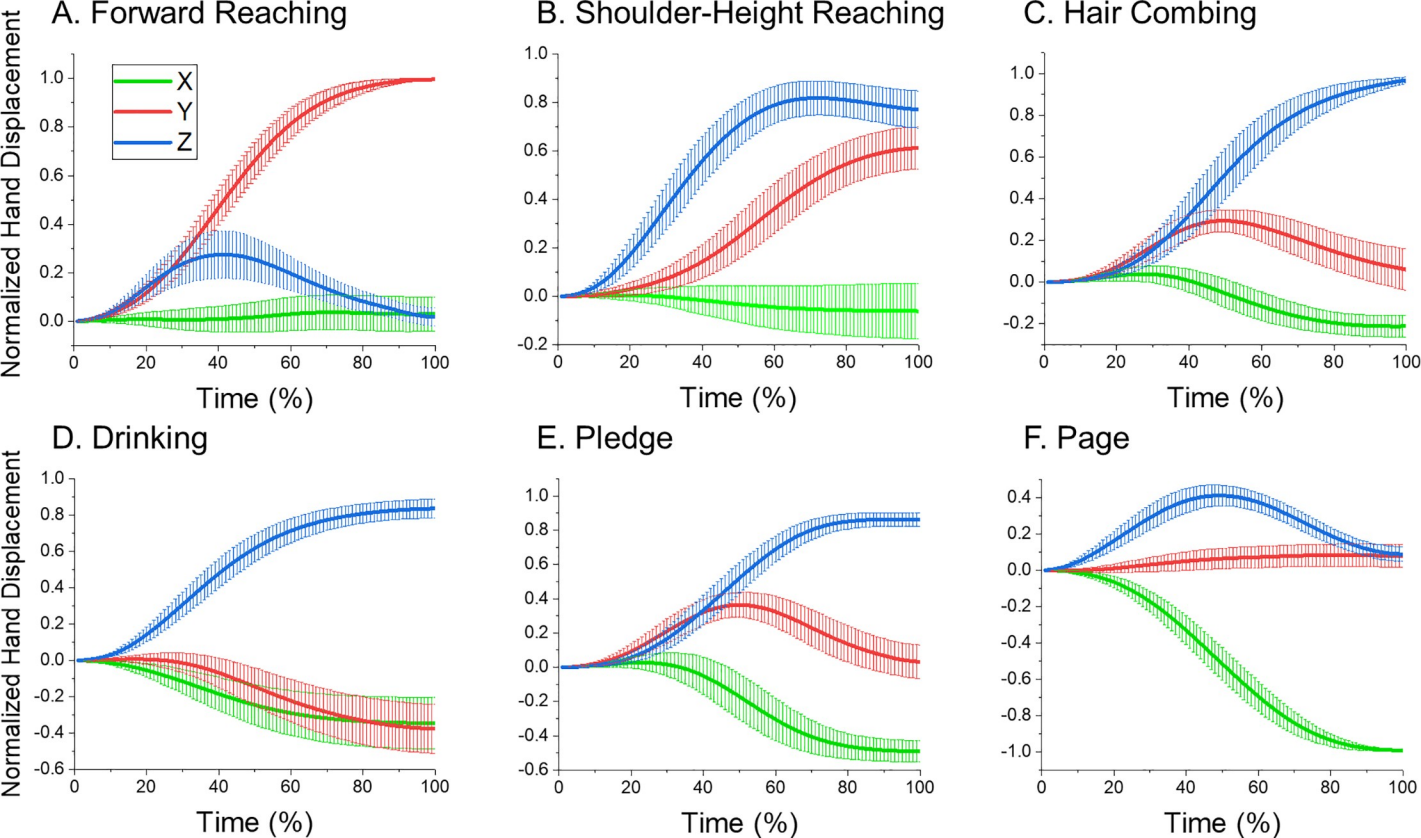
Means and SD of normalized hand displacements along the X, Y, Z coordinates of the global coordinate system. The normalization was applied to each movement by shifting the system’s origin to the initial hand position, re-scaling movement time to 100%, and dividing the displacement along each axis at each moment of time by the distance between the initial and final hand location. The x-values increased when the hand moved away from the body, the y-values increased when the hand moved to the right from the body, and the z-values increased when the hand moved up.

Trajectory directions and shapes also varied across the tasks, which follows from the distinct combinations of the hand displacements along the X-, Y-, and Z-axes of the global coordinate system in each task (Fig 3). Since different subjects initiated movements in different space locations and moved with different speeds and to different distances, the averaged results in Fig 3 were obtained after normalization of the displacements by the initial position of the hand, movement time, and the distance between the initial and final location of the hand, as detailed in the figure legend. Fig 3 shows that the hand trajectory represented a unique combination of motions along the 3 axes for each task. The limited SD values suggest that the normalized displacements of the hand were consistent across subjects in each task. To verify the distinction of the 6 tasks, K-means clustering performed in Matlab was applied to the amplitudes of the hand motion along the X-, Y-, and Z-axes obtained in all trials combined across subjects. The mean and SD of these amplitudes are shown in Fig 3 at 100% of time. Seven distinct clusters were revealed with the trials of each task representing a separate cluster. An exception was shoulder-height reaching the trials of which were divided in 2 clusters (20 and 22 trials, respectively), with the first cluster being characterized by lower y-values compared with the second cluster. The only classification error was a single drinking trial that was mis-classified as a pledge trial.

### DOF kinematics

The role of the 7 DOF in transporting the hand for each task can be deduced from DOF displacement amplitudes and contributions to hand velocity shown in Table 1. The amplitudes show that the shoulder flexed substantially (the mean was higher than 20° in all 6 tasks). Another consistently used DOF was elbow flexion/extension (the mean was higher than 20° in 5 tasks. The other shoulder and elbow DOF were substantially used in some tasks only.

**Fig 4:**
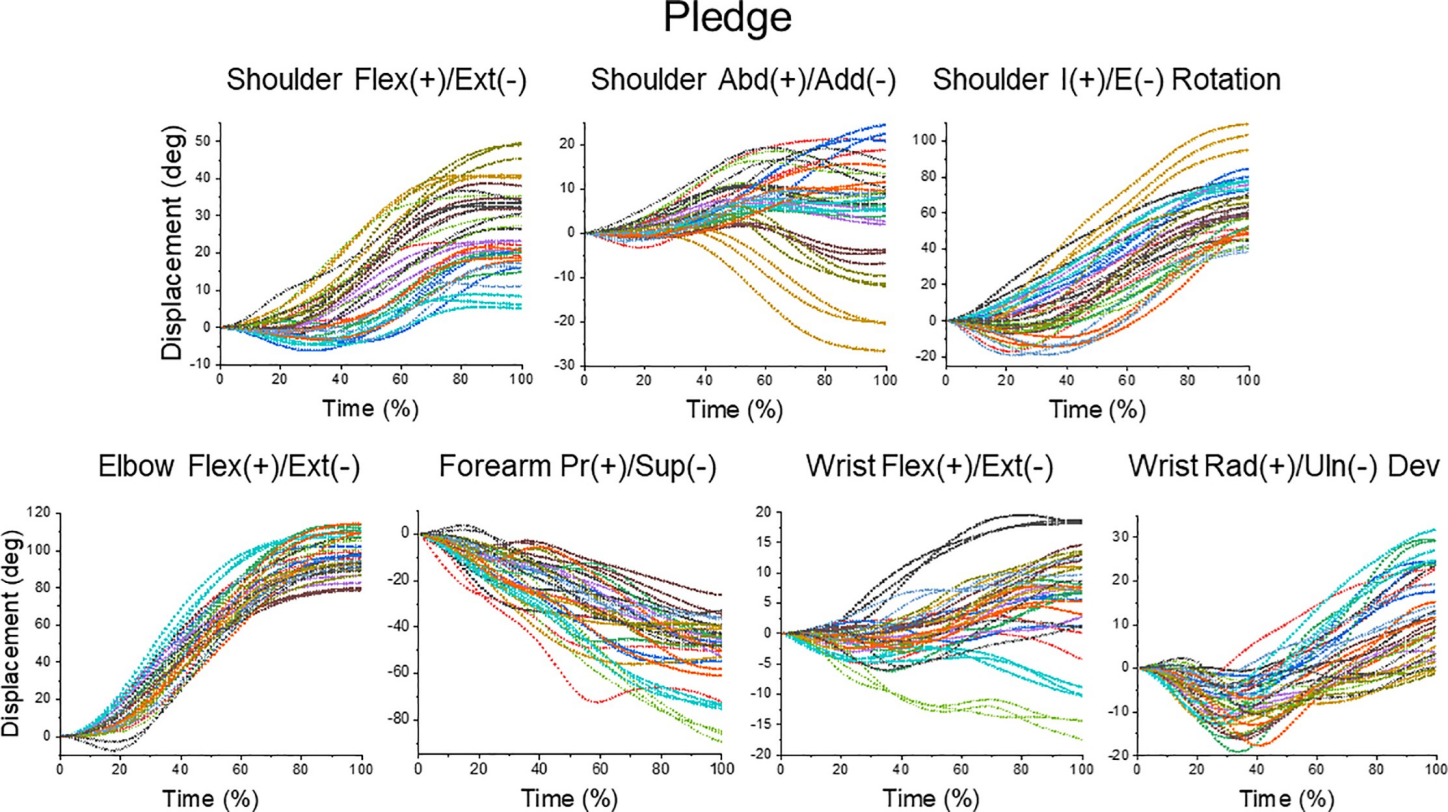
Individual displacements of the 7 DOF during all pledge movements. Each color shows 3 curves representing DOF displacements during the 3 trials performed by each subject. The 3 curves of the same color often had the same shape and amplitude, suggesting that at least some subjects used the same pattern of DOF motions during different trials of the task. The patterns of DOF motions differed across subjects.

It is noteworthy that SD of the DOF amplitudes were often large (Table 1). Since SD was computed across all trials of each task, the high values of SD could be a result of both inter- and intra-subject variability. Fig 4 provides insights about variability of each type. It shows individual DOF displacements during pledge with the 3 trials performed by each subject presented with the same color. The curves of the same color cluster together and have the same shape at least for some colors. Also, the curves of different colors noticeably vary in shape and amplitude. The same observations can be made for the other 5 tasks (the figures are provided as supplemental materials [53]. These observations suggest that the pattern of DOF motions varied across subjects and it was often preserved in the three trials performed by each subject. To quantify these observations, we computed individual intra-subject variability as SD of the DOF displacement values obtained at the end of the 3 movements (at 100% time) in each task. We also computed the inter-subject variability as SD across subjects of the individual means across the 3 trials of the final DOF displacements. The t-test revealed that for each task and each DOF, the individual values of intra-subject variability were significantly different from the inter-subject variability (P < 0.001 for all DOF in all tasks). Table 1 includes the minimal and maximal values of a ratio between the inter-subject variability and mean intra-subject variability. This ratio was higher than 1 for all DOF in all tasks, supporting the visual observation from Fig 4 that inter-subject variability was higher than intra-subject variability.

**Fig 5:**
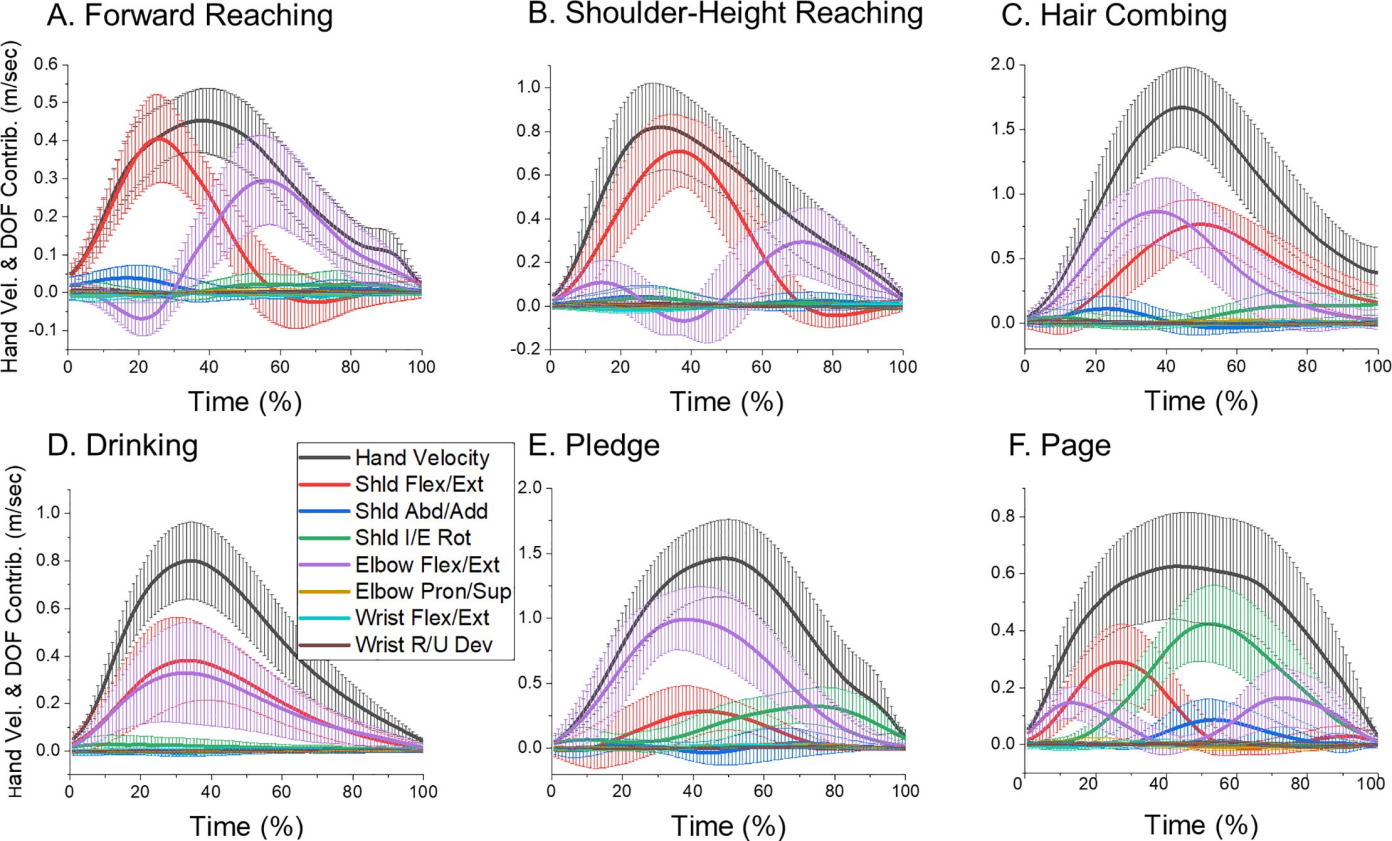
Means and SD of the hand velocity and the contributions of each DOF to hand velocity in each task. The data were averaged across all movements performed by all subjects for each task.

The percentage of DOF contributions to the hand velocity shown in Table 1 and actual contribution values shown in Fig 5 demonstrate the role of each DOF in transporting the hand in space. Consistent with the results for DOF amplitudes, shoulder and elbow flexion/extension played an important role in transporting the hand during all tasks. However, phasing and relative contribution of shoulder and elbow flexion/extension varied across the tasks (Fig 5). During both reaching tasks (Fig 5A and 5B), the 2 DOF contributed consecutively, first the shoulder and then the elbow. Both DOF started to contribute at the movement beginning during hair combing, drinking, and pledge (Fig 5C–5E). Elbow flexion/extension contribution had 2 peaks during page turning (Fig 5G), at the movement beginning and end. Thus, even though these 2 DOF played cardinal roles in transporting the hand in different directions, each direction required a unique combination of their contributions.

Shoulder internal/external rotation was also influential during some of the tasks, specifically pledge and page turning. The contribution to the hand velocity of shoulder abduction/adduction was minor during all tasks (the mean value was less than 8%), even when its amplitude was substantial. For example, this DOF amplitude was 33° during hair combing while its hand velocity contribution was only 3%. Apparently, the shoulder was abducted during this task to provide the arm’s posture that was required to orient the comb horizontally and move it above the head. The other 3 DOF provided negligible contribution to hand velocity (2% or less) even when the DOF amplitude was noticeable. For example, the forearm was substantially pronated during page turning and supinated during pledge and hair combing. Amplitude of the 2 wrist DOF was also noticeable during some tasks. However, the contribution of these DOF to hand velocity was near zero during all tasks. These results suggest that the role of forearm pronation/supination and the two wrist DOF was mainly to orient the hand in space as required by the task, and not to transport the hand.

The DOF contributions to hand velocity provided other important information. Namely, they allowed us to estimate validity of our kinematic model of the arm. This model is prone to errors due to a number of factors, including the DOF definition, marker placement for movement recording, estimation of the arm’s segment lengths, and trunk motion. Significant errors in the model would result in a substantial difference between the sum of the 7 DOF contributions and the actual hand velocity. The bottom row in Table 1 shows that the average percentage of the actual hand velocity accounted for with the sum of the DOF contributions was between 90% and 97% across the tasks. Taking into account the multiple factors that can reduce the model accuracy, these numbers provide a satisfactory validation of the arm model, and thus, our results.

### Joint control

As presented in Methods, the model used to compute torques at the three arm joints included a stationary trunk. We verified this assumption by computing the range of motion of the RA marker. The mean value across all 6 tasks was 2.6cm (SD = 1.2cm) in the anterior-posterior direction and 2.1cm (SD = 1.1cm) in the lateral direction. We assumed that this motion was small enough to be neglected. In support of this assumption is that when trunk motion was disregarded, the contributions of the 7 DOF of the arm accounted for more than 90% of the hand velocity. It is also noteworthy that the results presented next are highly robust because we do not analyze actual torque values. Our results mainly rely on comparisons of torque signs, and therefore, have minimal sensitivity to errors in torque computations that may arise due to small shoulder motion and inaccuracies in estimation of the anthropometric characteristics.

**Fig 6:**
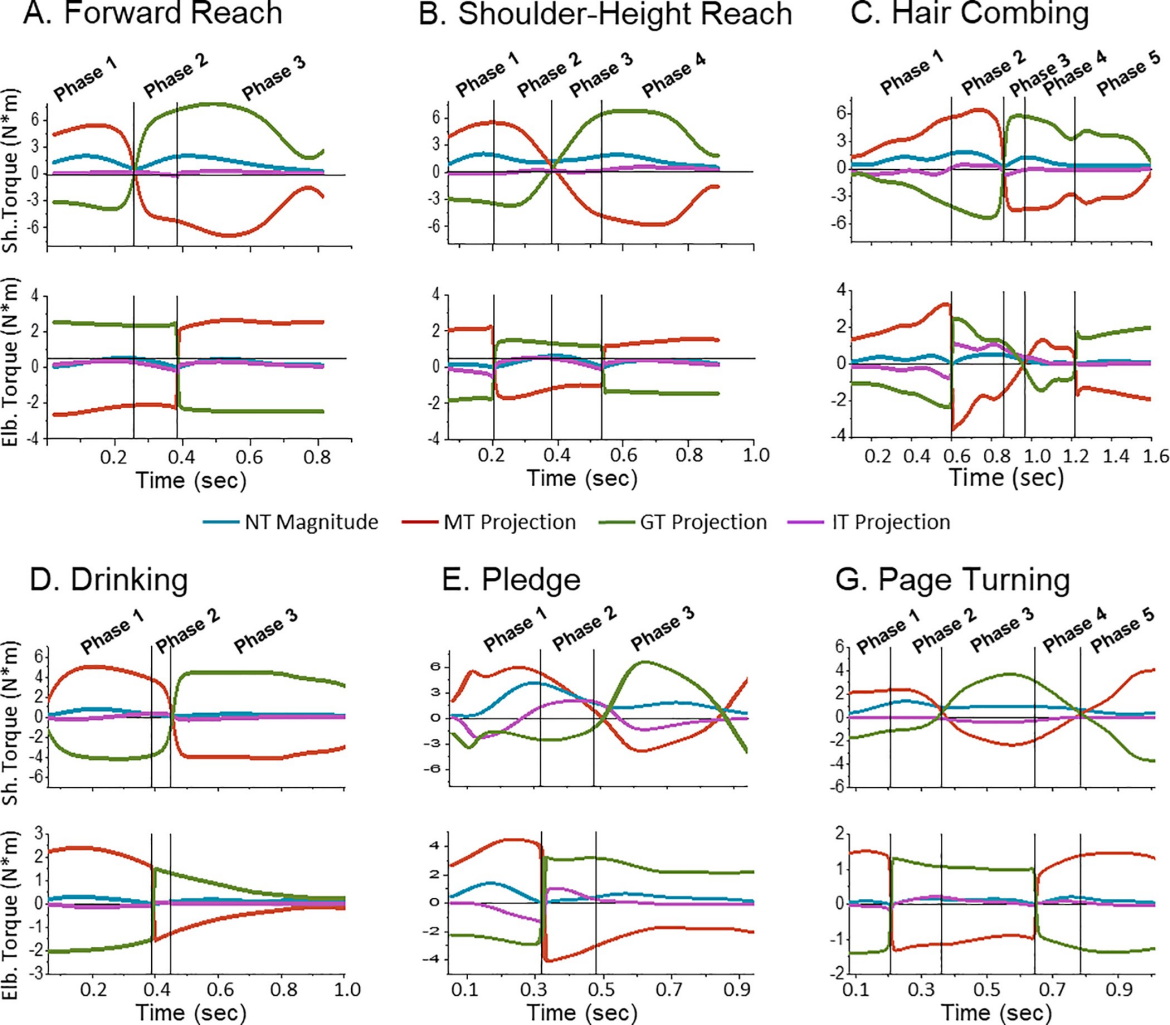
Individual examples of projections of MT, GT, and IT on NT at the shoulder and elbow during performance of the 6 ADL tasks by a representative participant. The blue curves show the magnitude of **NT**. See the definition of the movement phases in the text.

To establish control of each joint, we examined the role of **MT** and **PT** = **GT** + **IT** in the generation of **NT**. This was achieved through analysis of the contribution of the **MT**, **GT**, and **IT** projections on the **NT** vector. Since the kinetic analysis revealed that the wrist DOF provided little contribution to the hand translation in space, the torque analysis is reported first for the shoulder and elbow and then separately for the wrist. Fig 6 shows individual examples of the **MT**, **GT**, and **IT** projections and **NT** magnitude at the shoulder and elbow during a single trial of each task performed by a representative participant. In all tasks, the **GT** projection on **NT** was much higher than the **IT** projection, which shows that **GT** predominantly determined **PT**. Also, the **GT** and **MT** projections had opposite signs. **NT** at the shoulder and elbow was therefore generated either actively, by **MT**, or passively, by **PT**, while the other torque resisted the generation of **NT**.

We considered movement phases based on the combinations of active and passive generation of NT at the shoulder and elbow joints. Each phase was characterized by one of the 4 modes of shoulder/elbow control: “both-active” when **NT** at both joints was produced by **MT** with **PT** opposing it, “shoulder-led” when **NT** was produced at the shoulder by **MT** with **PT** opposing it and at the elbow by **PT** with **MT** opposing it, “elbow-led” when **NT** was produced at the shoulder by **PT** with **MT** opposing it and at the elbow by **MT** with **PT** opposing it, and “both-passive” when **NT** at both joints was produced by **PT** with **MT** opposing it. MTC was used to define the phase boundaries. Due to the opposite signs of **MT** and **GT** projections and the dominance of **GT** in **PT**, MTC was predominantly near 1 when **NT** was generated by **MT** and 0 when **NT** was generated by **PT**. Only phases that were greater than 5% of movement time and 5% of the hand trajectory length were considered. The phase boundaries were determined as the time moments when MTC crossed the value of 0.5 either at the shoulder or elbow. At both joints, mean MTC (averaged within each phase, then across the trials performed by each subject, and then across subjects) was higher than 0.95 (SD <0.01) when MT produced NT, and it was lower than 0.06 (SD < 0.02) when PT produced NT. An exception was pledge during which the shoulder-led phase was characterized by MTC = 0.8 (SD = 0.2) at the shoulder.

**Fig 7:**
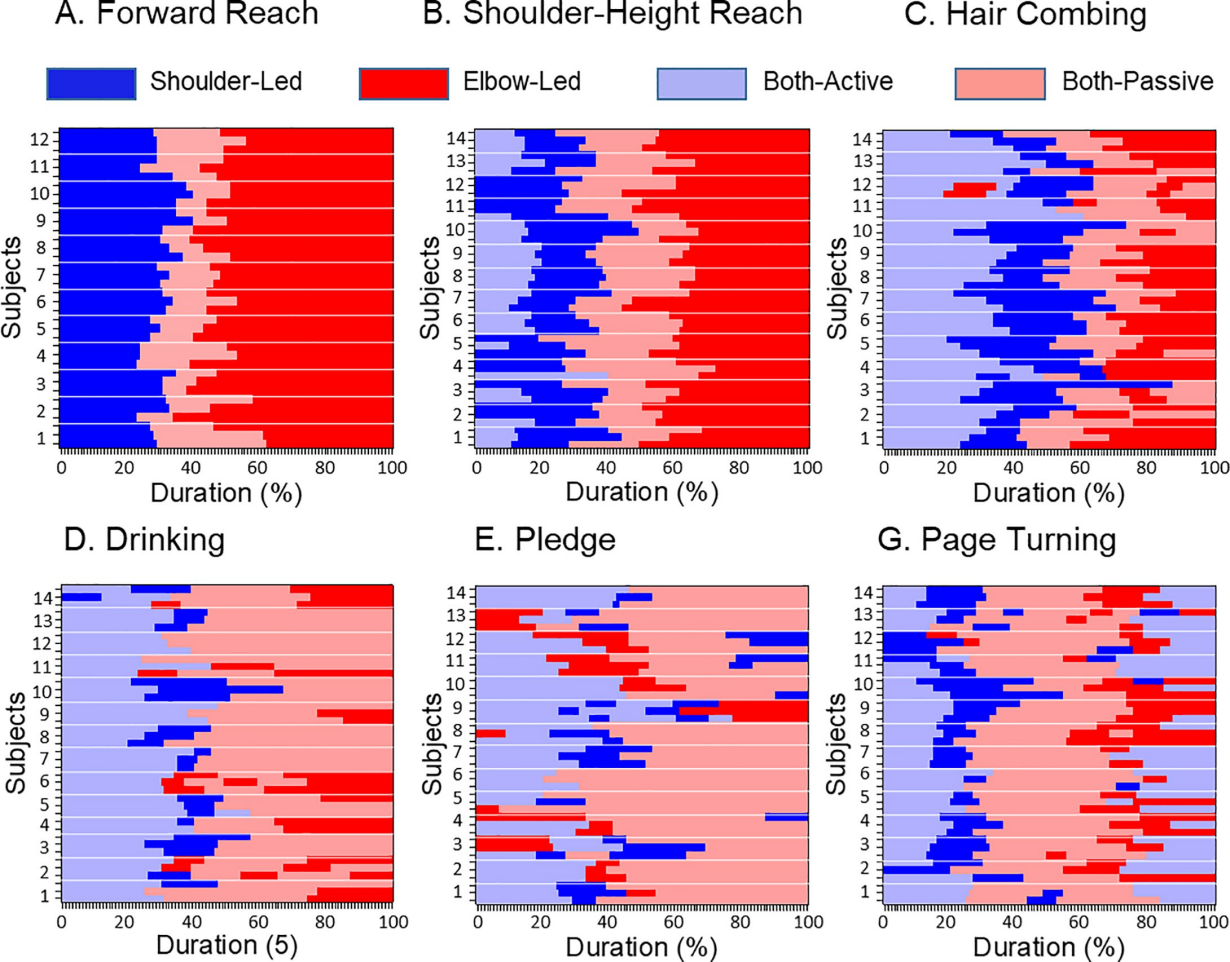
The sequence and relative durations of the 4 joint control phases during each trial for the 6 ADL tasks. The horizontal white lines separate trials performed by each subject. While each of the 14 subjects performed 3 trials in the majority of the tasks, data from 12 subjects (3 trials from each subject) were available during forward reaching and only 2 trials were available from subject 4 during drinking.

Fig 7 presents relative duration of each phase in each trial across subjects. The both-active control mode was typically observed during movement initiation in all tasks except for reaching forward. The remaining portions of the movements were performed with the use of the other 3 modes of control, during which either one or both joints were rotated passively. An exception was page turning which included a second both-active phase at the movement end. The mean portions of movement time and trajectory length covered during the both-active phases across all trials of all subjects in which this phase was included are presented in Table 2. The both-active mode covered less than 50% of movement time and trajectory length in all tasks. A t-test applied to these percentage data across all trials (in all subjects) that included the both active phase confirmed that both were less than 50% (P < 0.001 for each task both for the percentage of movement time and trajectory length).

**Table 2:** Mean and SD of phase characteristics in the phase sequence most frequently used for each movement type.

	Phase 1	Phase 2	Phase 3	Phase 4	Phase 5
**Forward Reaching**					
Phases (3 total)	Shoulder-Led	Both-Passive	Elbow-Led		
Time (%)	26 (10)	16 (8)	46 (17)		
Tr. Length (%)	28 (12)	25 (7)	44 (11)		
Sh MTC; Elb MTC	1.0 (0); 0.0 (0)	0.0 (0); 0.0 (0)	0.0 (0); 1.0 (0)		
**Shoulder-Height Reaching**					
Phases (4 total)	Both-Active	Shoulder-Led	Both-Passive	Elbow-Led	
Time (%)	17 (8)	23 (3)	24 (7)	39 (8)	
Tr. Length (%)	15 (12)	34 (7)	33 (6)	24 (3)	
Sh MTC; Elb MTC	1.0 (0); 1.0 (0)	1.0 (0); 0.0 (0)	0.0 (0); 0.0 (0)	0.0 (0); 1.0 (0)	
**Hair Combing**					
Phases (4 total)	Both-Active	Shoulder-Led	Both-Passive	Elbow-Led	
Time (%)	32 (7)	20 (7)	17 (6)	25 (7)	
Tr. Length (%)	28 (8)	34 (9)	22 (7)	16 (5)	
Sh MTC; Elb MTC	1.0 (0), 1.0 (0)	1.0 (0), 0.0 (0)	0.0 (0), 0.0 (0)	0.0 (0), 1.0 (0)	
**Drinking**					
Phases (3 total)	Both-Active	Shoulder-Led	Both-Passive		
Time (%)	29 (8)	12 (8)	45 (12)		
Tr. Length (%)	32 (6)	22 (13)	40 (10)		
Sh MTC; Elb MTC	1.0 (0), 1.0 (0)	1.0 (0), 0.1 (0)	0.0 (0), 0.0 (0)		
**Pledge**					
Phases (3 total)	Both-Active	Elbow-Led	Both-Passive		
Time (%)	29 (6)	25 (11)	42 (10)		
Tr. Length (%)	21 (8)	21 (8)	53 (10)		
Sh MTC; Elb MTC	1.0 (0), 1.0 (0)	0.8 (0.2), 0.1 (0)	0.1 (0.1), 0.0 (0)		
**Page Turning**					
Phases (5 total)	Both-Active	Shoulder-Led	Both-Passive	Elbow-Led	Both-Active
Time (%)	17 (4)	12 (5)	38 (6)	12 (5)	18 (4)
Tr. Length (%)	10 (4)	15 (5)	54 (5)	14 (7)	9 (3)
Sh MTC; Elb MTC	1.0 (0), 1.0 (0.1)	1.0 (0), 0.0 (0)	0.0 (0), 0.0 (0)	0.0 (0), 1.0 (0)	1.0 (0), 1.0 (0)

The phase characteristics include movement time percentage, trajectory length percentage, and shoulder and elbow muscle torque contribution (MTC).

The emergence of the both-active phase at the beginning of the tasks that required lifting the hand to a considerable height suggests that the purpose of this phase was to overcome gravity. We tested this by computing the portion of duration of this control mode during which the vertical component of the hand acceleration was positive (accelerating while moving up or decelerating while moving down). This portion averaged across trials of all subjects was 0.99 (SD = 0.06) during shoulder-height reaching, 0.98 (SD = 0.05) during hair combing, 0.84 (SD = 0.19) during drinking, 0.95 (SD = 0.16) during pledge, and 0.90 (SD = 0.12) during page turning. These results demonstrate a strong association of the both-active control mode with the need to overcome gravity.

Fig 7 shows that the sequences of the other phases could be different in different trials of the same task, especially during drinking and pledge. Table 2 represents the phase sequences that were observed most frequently in each task. Fig 7 compliments the finding obtained from Fig 4 that at least some subjects tended to use the same pattern of DOF motions across trials. For example, the trials that did not include the both-active mode during shoulder-height reaching were not distributed across subjects, but were repetitively performed by the same subjects. During drinking, the major sequence was observed in all 3 trials of subjects 3, 5, 7, 8, 10, 14, while the other subjects rarely used this sequence. During pledge, all 3 trials of subject 9 only were performed with two shoulder-led phases interrupted by the both-active phase. These examples show that in addition to the consistency of DOF motions observed in Fig 4, there was a tendency to use the same pattern of joint control at least for some subjects, and there was a variability in this pattern across subjects.

The wrist was not included in the above analysis because the pattern of wrist control was highly variable across subjects, trials, and even within each trial. This pattern included frequent changes between active and passive control. This is typical of wrist control due to stabilization of this joint through suppression of **PT** with **MT** [17, 22, 36]. The changes between active and passive control were independent of the shoulder and elbow control phases, which suggested independence of control of hand orientation from control of the shoulder and elbow DOF responsible for translation of the hand in space.

### Joint control during returning movements

The kinetic analysis demonstrated that the arm movements from the target back to the initial position were performed largely symmetrically to the movements toward the targets. Accordingly, the main sequences of the joint control phases were opposite to those observed during movements to targets and shown in Table 2. In particular, the both-active phase was the last during shoulder-height reaching, hair combing, drinking, and pledge, and it was usually observed twice during page turning, at the movement beginning and end. Notably, there were returning movements of each type that did not include the both-active control mode at all (34 forward reach, 5 shoulder-height reach, 5 drinking, 4 hair combing, 9 pledge, and 1 page turning). The mean and SD of the portions of the movement duration and distance covered with the both-active control mode were, respectively, 18.7% (9.6%) and 12.4% (6.2%) during shoulder-height reach, 20.9% (9.7%) and 22.1% (11.3%) during hair combing, 31.6% (13.3%) and 31.0% (15.4%) during drinking, 21.9% (15.3%) and 22.2% (17.3%) during pledge, and 31.5% (11.5%) and 21.9% (7.2%) during page turning. Like in the movements to the targets, in the returning movements that included the both-active phase, this phase covered less than 50% of movement duration and trajectory length (P < 0.001 in all tasks).

## Discussion

Activities of daily living, such as eating, grooming, dressing, and cooking, require the ability to perform arm movements in different directions, coordinating motion at multiple DOF in a specific way related to each task. Understanding control and coordination of the arm’s joints that result in natural arm movements is necessary for assessments of decline in the ability to perform ADL caused by aging and motor disorders, and development of effective rehabilitation approaches. Here we examined whether the simple trailing joint control pattern dominates performance of natural arm movements despite their biomechanical complexity. Six tasks simulating ADL were selected to represent movements in different directions: toward, away from, and to the other side of the body at different heights. Since during all 6 tasks the hand initially moved upward, we also analyzed the returning movements, thus including 6 different downward movement directions.

Our results confirm that the hand movement varied across the 6 tasks, each involving a specific combination of motions along the 3 spatial coordinates. The hand was transported in space mainly through shoulder and elbow flexion/extension, although the contributions of these 2 DOF were unique for each task. Shoulder internal/external rotation also contributed during pledge and page turning, i.e., when the task included large lateral translation of the hand. The primary role of the other 4 DOF was to orient the hand according to task requirements.

Shoulder and elbow control responsible for generation of hand motion was determined through the analysis of contributions of active control represented by MT and passive effects represented by PT = GT + IT to the generation of NT. We address joint control as active when MT generated NT and suppressed PT (MTC ~ 1) and as passive when NT was generated by PT and MT resisted NT (MTC ~ 0). It is noteworthy that although GT and MT were much higher in magnitude than IT, the contribution of IT to NT was comparable to those of MT and GT because only small portions of MT and GT were used for movement production. Active joint control was provided by a small portion of MT, while the biggest portion of it compensated for resistive GT. During passive control, a small portion of GT was used to generate NT while the entire MT was used to compensate for the biggest portion of GT.

### Dominance of the trailing pattern in joint control

During all studied ADL tasks, the dominant portion of each movement was produced by rotating passively either the elbow (shoulder-led mode) or shoulder (elbow-led mode) or both (both-passive mode). These three modes represent the trailing joint control pattern characterized by the usage of PT for rotation of one or both joints. The obtained results therefore support our hypothesis that the trailing pattern dominates control of daily arm movements. Since previous studies of joint control mostly focused on horizontal arm movements, the shoulder- and elbow-led control modes were typically observed. The both-active mode was reported in a study of 3D arm movements [31]. This mode was revealed during downward movements. Our results suggest that the both-passive mode is common during 3D movements, whether the hand moves up or down. Indeed, gravity can be the major cause of deceleration of the limb during upward motion and acceleration during downward motion.

The both-active mode during which both joints were moved actively, and thus, the trailing pattern was not used, was observed in 5 out of the 6 tasks. However, it constituted a minor movement portion (significantly less than 50%). Specifically, the longest use of the both-active control mode was during page turning in phases 1 and 5 that together provided, on average, 17% + 18% = 35% of movement duration. The longest portion of the trajectory length covered with the both-active mode was 32% during drinking.

The emergence of the both-active mode at the beginning of the movements that lifted the hand to a substantial height suggests that this mode was used to overcome gravity at movement initiation. This interpretation is supported by the finding that during the dominant portions of the both-active phases (84%-99%) in all tasks, the hand was accelerated upward. The emergence of the both-active phase at the end of the returning movements also supports this interpretation because the limb moved downward, and gravity had to be compensated for to terminate limb motion.

Notably, there was no direct dependence of the duration of the both-active mode on the height to which the hand was lifted. For example, this portion was substantially smaller during the shoulder-level reaching than pledge, even though the hand moved higher in the former task than the latter. Another factor that could influence the movement portion covered by the both-active phase was the joint coordination pattern. Indeed, this portion was larger during the tasks that required flexion of both the shoulder and elbow, including drinking, hair combing, and pledge, and it was shorter during the 2 reaching tasks that were performed with shoulder flexion and elbow extension. Previous analyses of horizontal arm movements demonstrated that IT assists rotation of both the shoulder and elbow during movements involving flexion at one joint and extension at the other joint, and IT resists rotation of both joints if both of them simultaneously flex or both extend [12, 20].

The effect of GT on joint rotation also depended on the joint coordination pattern. While the shoulder had to overcome GT to accelerate into flexion during all tasks, GT assisted acceleration of the elbow into extension during the 2 reaching tasks and GT resisted acceleration of the elbow into flexion required for drinking, pledge, and hair combing. During the deceleration phase, the effect of GT on the 2 joints was opposite: GT assisted shoulder flexion during all tasks, resisted elbow extension during the reaching tasks, and assisted elbow flexion during drinking, pledge, and hair combing. Thus, IT and GT provided a synergistic effect on elbow acceleration during most of the tasks.

Despite the need to simultaneously accelerate both joints into flexion during drinking, pledge, and hair combing, and thus, overcome PT = IT + GT with MT, the both-active phase accounted for only about 30% of the task. One way of shortening the both-active phase could be by delaying the initiation of flexion at one joint with respect to the other joint. Fig 5 shows that the only task during which the shoulder and elbow DOF started to contribute to the hand velocity simultaneously was drinking. Contribution of shoulder flexion/extension was delayed during hair combing and pledge. The delay in flexion of the shoulder relative to the elbow that allowed IT and/or GT to assist the joint motions was reported in a study in which the hand was lifted up to catch a ball [39]. The arm movement was performed in the sagittal plane by 2 groups of 10 year old boys, typically developing and with a developmental coordination disorder (DCD). The arm freely stretched down in the initial position, and a ball was caught slightly above the shoulder level. Typically developing children demonstrated skillful performance of the task, catching 85% of the balls, while children with DCD had only 32% success rate. The task required flexion of the shoulder and elbow. It was found that typically developing children performed the movement with a sequence of active flexion first of the elbow and then the shoulder, while NT at the other joint was generated passively. Joint coordination was different in children with DCD, who tended to actively flex the shoulder and elbow simultaneously.

Our findings in combination with the results for catching movement [39] support the prevalence of joint control that involves predominantly passive motion of at least one joint of the arm. They also suggest that the constraints of the task may make the both-active phase unavoidable. In particular, this phase is often necessary to overcome the initial resistance of gravity when the hand needs to be lifted to a substantial height.

Only 6 ADL tasks were included in the current study. The tasks were not completely natural. For example, trunk motion was not allowed and the initial arm position was the same for each task set. It is possible that in natural conditions, we orient the trunk to increase the use of the trailing pattern. For example, having a glass of water in front of the trunk and not in front of the shoulder may decrease the both-active phase during drinking. Future research should investigate if preferred trunk orientations support the use of the trailing pattern for control of arm movements. Another limitation of the present study was the availability of 3 trials of each task only. Nevertheless, the studied movements were diverse, differing in movement directions and combinations of rotations of the arm DOF, and they were performed under minimal spatial and temporal restrictions. This allows us to expect that the 4 phases revealed here are commonly observed during natural arm movements. The dominance of the trailing pattern and the use of the both-active control mode mainly for overcoming gravity are robust findings because they were observed in all tasks during both movements to the targets and returning movements.

Although only discrete movements were analyzed in this study, the symmetrical structure of the returning movements allows predictions for the organization of joint control during cyclic movements. The phase sequences were reversed during the returning movements, which suggests that the last phase of the preceding movement component would be merged with the first phase of the reversal component if the movements were produced cyclically. This expectation is supported by knowledge that during single-joint movements, the two distinct bursts of the muscle that decelerate preceding movement and accelerate the reversal movement are merged in a single, prolonged burst during cyclic movements. The difference between discrete and cyclic movement control was specified as nullification of both velocity and acceleration at the end of discrete movements as compared with nullifying velocity only at the reversals of cyclic movements [54]. While the same muscles provide the deceleration and successive acceleration of the limb during discrete and cyclic movements, these muscles would need to work harder during discrete movements to dissipate movement energy at the end of the first movement and to accumulate movement energy at the beginning of the returning movement. However, joint control patterns would be similar during discrete and cyclic movements.

The different function of the wrist (orientation of the hand in space) compared with the shoulder and elbow that transported the hand, and the frequent changes between the joint control modes at the wrist that were not related to the mode changes in the proximal joints, suggest that wrist control was largely independent from shoulder and elbow control. Indeed, the tasks required specific orientation of the wrist in space rather than coordination of motion between the wrist and the proximal joints. The independent wrist control was possible because the mechanical effects that depend on motion of the proximal joints were largely suppressed by MT. Suppression of passive effects may not require a significant neural effort because it may be produced to a large extent (especially during well-learned movements) at the spinal level by the neural circuitries that process somatosensory feedback, including the stretch reflex-mediating circuitry and muscle synergies producing co-activation of antagonistic muscles [55].

### Theoretical implications for neural control of multi-joint movements

What can be the reason for the prevalence of the trailing pattern during daily arm movements? One possibility is that this prevalence is a natural consequence of the mechanical properties of the limb and environment that cause interaction and gravitational torques. However, previous research provides strong evidence against this interpretation. First, it was demonstrated that the trailing pattern is a feature of skillful movements [14–16, 37], which suggests that it emerges as a result of motor learning. Second, when the task allowed a selection of movement direction (and thus, joint coordination pattern), participants preferred the directions in which the trailing pattern could be used [19, 35]. Third, studies of the effect of various motor deficiencies on joint control consistently reported deviations from the trailing pattern [2, 3, 5, 38–42]. These findings make it clear that the biomechanical properties of the limbs, which are the same in novices and experts, and healthy people and patients with neurological disorders, do not dictate the use of the trailing pattern. Rather, these findings point to optimality of the trailing pattern. While testing optimality of the trailing pattern is out of the scope of the present study, our results are consistent with the interpretation of the trailing pattern as optimal because the daily movements of healthy young adults studied here represent nearly optimal joint coordination.

If this interpretation is correct, what criterion does the trailing pattern optimize? One of the most frequently discussed optimality criteria is minimization of muscle effort for movement production [56–58]. This criterion suggests that passive torques are used during the trailing pattern to reduce muscle effort. This interpretation was questioned by a finding that when the task allowed subjects to choose a movement direction, they demonstrated preferences for directions that included the direction of the biggest arm inertia [19] and the upward direction [31] which required higher muscle effort than most of the non-preferred directions. However, NT at either the shoulder or elbow was generated passively in all preferred directions in those studies.

An alternative interpretation is that the trailing pattern represents a control strategy that reduces neural effort for joint coordination. The understanding that cognitive resources are limited and they need to be used optimally underlies many recent models and theories of perception, information processing, decision-making, memory, reasoning, attention, and other phenomena of cognitive psychology (for review, see [59]). This insight is relevant to motor control that includes most of the listed cognitive functions [60]. Accordingly, it was suggested that minimization of neural computations involved in movement planning and execution is an essential component of the movement control strategies [61]. Experimental evidence suggesting that the trailing pattern reduces neural effort for movement control was provided [35]. In that study, the motor task required a frequent use of MT for generation of NT simultaneously at the shoulder and elbow. When a secondary cognitive task was added, subjects changed performance of the motor task in a way that allowed them to mainly use the trailing pattern. In addition to the experimental evidence, it was theoretically justified that the trailing pattern minimizes the amount of information that the brain needs to process for joint coordination [34]. The interpretation that the trailing pattern reduces cognitive load of joint coordination is consistent with our finding that the trailing pattern is dominant during daily arm movements because these movements are secondary to the goals of daily activities, which are usually cognitive. For example, during setting a table for dinner, we focus on what items need to be placed on the table and how they should be arranged, and not on how we coordinate shoulder and elbow motions.

Whatever the advantage of the trailing joint control pattern is, the dominance of it during natural arm movements is consistent with the interpretation that mechanical properties of the limbs and environment are used for movement production. Since the trailing pattern is a feature of skillful movements [16, 37], the skillful use of the mechanical properties of the multi-segmental limbs likely emerges through evolution, motor and cognitive development, and motor learning as a result of adaptation to the environment (including limb biomechanics) and cognitive constraints.

### Practical applications of the obtained findings

Understanding optimal joint coordination is important for evaluation of joint dyscoordination caused by motor disorders. We propose that dyscoordinated arm movements are movements characterized by decreases in the use of passive effects for joint rotation. This definition of dyscoordinated movements is supported by deviations of shoulder and elbow control from the trailing pattern revealed in individuals after stroke [2, 5], Parkinson’s disease patients [3, 40], patients with deafferentation [41], children with DCD [39], and for movements of the non-dominant arm in healthy individuals [38, 42]. The trailing pattern enables quantification of these changes. For example, deviations from the trailing pattern observed in some of those studies was quantified by decreases in MTC at the leading joint, increases in MTC at the trailing joint, and decreases in the movement portion during which the trailing pattern was observed. It is possible that the characteristics of the sequences of the 4 control phases presented in Table 2 can be used as normative characteristics of coordinated performance of the studied ADL tasks, and deviations from these numbers would quantify dyscoordination, especially enlarging the duration of the both-active phase and trajectory portion covered by it. This hypothesis needs to be tested in future research through a comparison of joint control during daily arm movements in healthy subjects and patients with motor pathologies.

The recognition of the trailing joint control pattern as a dominant control pattern during skillful movements paves the way for enhancing rehabilitation and motor learning. Indeed, it was suggested that neurological motor disorders, including those caused by Parkinson’s disease, stroke, and DCD, reduce the ability to predict and exploit PT for movement performance [5, 39, 40, 62]. These patients often co-activate antagonistic muscles, which results in stiffer joints. Muscle co-activation may, at least partially, be a result of adaptation to the disorder because joint stiffness dampens passive effects, thus reducing the need for feedforward control, and also makes movements slower, thus allowing the reliance on feedback. Our results show that PT is used for rotation of the arm’s joints in a specific way in each movement direction. A promising approach to improve the ability to predict and use PT in motor disorders would therefore be performance of movements in different directions as fast as possible (to increase IT and reliance on feedforward control) with the instruction to maximally relax muscles and de-emphasize accuracy (to decrease reliance on feedback).

The emphasis on the trailing pattern may crucially benefit motor learning. If the trailing pattern underlying performance of a given movement is known, it can be easily verbalized by describing which joint should be used as leading, what mechanical effect it needs to generate, and how the other joints should use this mechanical effect. For example, a horizontal movement of the right arm from the shoulder along the left diagonal involves shoulder flexion and elbow extension. However, only the shoulder is actively rotated while elbow muscle activity is low, with the elbow joint rotated mainly due to passive mechanical effect of the shoulder motion [12]. When training a patient to improve performance of this movement, an instruction “focus on moving your shoulder and relax the elbow” may be more fruitful than the instruction “flex the shoulder and extend the elbow”, because the later can be produced through different patterns of joint control as movements in special populations demonstrate [40]. This example shows that formulating instructions for performing movements in terms of the actuation of various joints may be advantageous.

Knowing the underlying trailing joint control pattern may enhance learning of sports movements. There are sports movements for which the trailing pattern has been established intuitively and used for skill training. For example, novices in kayaking pull the paddle in water towards the body through shoulder extension and elbow flexion. This is inefficient and causes quick onset of shoulder muscle fatigue. Instructors indicate that paddling should be done through rotation of the trunk around its longitudinal axis, while both arms remain straight to transmit energy generated by the trunk to the paddle [e.g., see 63]. The trailing joint control structure used in many other sports remains unknown. Unravelling this structure may greatly improve effectiveness of training procedures. Also, the use of the trailing pattern suggests that during motor learning, attention should be focused on the leading joint and the mechanical effect it generates. Drawing attention to trailing joints may increase the active component of their control, which would be harmful for movement performance. This may be a reason why the internal focus of attention was often inefficient [64].

In conclusion, we examined joint control during 6 natural, unconstrained goal-directed movements with a redundant number of DOF. The movements were performed in different directions and through different combinations of motion in the arm’s DOF. Despite the variety and complexity of the movements, the trailing joint control pattern dominated during all of them. This finding supports the suggestion of previous studies [24, 42] that the dominance of the trailing pattern is a feature of skillful movements. We argue that the trailing pattern is a result of adaptation of control to the biomechanical complexity of multi-articular limbs and limitations of cognitive structures involved in movement control. The simplicity and transparency of the trailing joint control pattern are promising for quantitative assessment of dyscoordination in patients with motor disorders and development of movement performance instructions for rehabilitation of patients and improvement of motor skills in healthy people.
